# Draft Genome Sequence of Thermus sp. Isolate 2.9, Obtained from a Hot Water Spring Located in Salta, Argentina

**DOI:** 10.1128/genomeA.01414-14

**Published:** 2015-01-15

**Authors:** Laura E. Navas, Marcelo F. Berretta, Elio M. Ortiz, Graciela B. Benintende, Ariel F. Amadio, Rubén O. Zandomeni

**Affiliations:** a Instituto de Microbiología y Zoología Agrícola, Instituto Nacional de Tecnología Agropecuaria (INTA), Castelar, Buenos Aires, Argentina; b Estación Experimental Agropecuaria Rafaela, Instituto Nacional de Tecnología Agropecuaria (INTA), Rafaela, Santa Fe, Argentina; c Consejo Nacional de Investigaciones Científicas y Técnicas (CONICET), Buenos Aires, Argentina

## Abstract

Thermus sp. isolate 2.9 was obtained from a hot water spring in Salta, Argentina. Here, we report the draft genome sequence (2,485,434 bp) of this isolate, which consists of 11 scaffolds of >10 kbp and 2,719 protein-coding sequences.

## GENOME ANNOUNCEMENT

Bacteria belonging to Thermus genus have been isolated mostly from hot spring environments. Species from this genus contain relevant genes with potential biotechnological applications as sources of thermostable enzymes. There is also interest in studying the mechanism involved in bacterial adaptation to extreme natural environments. Thermus sp. isolate 2.9 is a Gram-negative aerobic, rod-shaped, thermophilic bacterium isolated from a hot water spring in Rosario de la Frontera, Salta, in the northwest of Argentina.

The draft genome sequence data were generated using a combination of the Roche 454 GS and Illumina MiSeq platforms, producing unpaired and paired-end reads, respectively, together with an optical map of the genome. The 454 FLX shotgun data (Macrogen, Seoul, South Korea) consisted of 215,557 reads achieving 32-fold coverage of the genome. First, Newbler was used to assemble those reads in 119 contigs of >1 kbp. The contigs were aligned against the optical map using MapSolver version 3.2 and also against the genomes of Thermus thermophilus HB8, T. thermophilus HB27, and Thermus aquaticus Y51MC23 using Mauve 2.3.1 (1). The analysis of the alignments allowed the ordering of contigs, and 32 gaps were closed by primer walking and sequencing of the PCR products over the gap, reducing the number of contigs to 87. To improve the genome assembly, an Illumina paired-end library was constructed using long jump distance technology with an insert size of 8 kbp (MWG Eurofins), generating 953,708 paired reads with an average length of 124 bp, which achieved 110-fold coverage. Finally, scaffolding and *in silico* gap filling were performed with SSCAPE 2.0 (2) and GapFiller 1.10 (3), respectively, resulting in 11 scaffolds of >10 kbp, containing 62 contigs. It represents an efficient strategy for sequencing genomes with a high G+C content and high proportions of repeated sequences, in which obtaining a single contig is unlikely (4).

The final assembly of Thermus sp. isolate 2.9 presented a total size of 2,485,434 bp, with an average G+C content of 67.29%. It was subjected to automated annotation using the RAST server 2.0 (5) and NCBI Prokaryotic Genome Annotation Pipeline (PGAP) (http://www.ncbi.nlm.nih.gov/genomes/static/Pipeline.html). The tRNA and rRNA genes were predicted using tRNAScan-SE 1.21 (6) and RNAmmer 1.2 (7), respectively.

Annotation by RAST predicted 2,719 protein-coding genes, 45% of which were assigned to 360 subsystems. Thermus sp. isolate 2.9 has one copy of 23S/5S and 16S rRNA genes and 46 tRNA genes. T. thermophilus HB8 (score, 529), HB27 (score, 526), and T. aquaticus (score, 424) were reported by RAST to be the closest neighbors of Thermus sp. isolate 2.9. Also, analysis using BLAST showed that three scaffolds, corresponding to 394,087 bp, have similarities with known plasmids of T. thermophilus strains.

From all enzymes identified by the annotation, 435 were selected for their potential applications in agriculture, biosensors, biotechnology, environment, energy, food, medicine, and other industries. In order to assess their functionality, the cloning and expression of some of these enzymes in Escherichia coli are under way. Genome-based knowledge of thermophiles is of great importance and interest for not only assessing the diversity of their enzymes but also understanding their metabolic adaptations to extreme living conditions.

### Nucleotide sequence accession numbers.

This whole-genome shotgun project has been deposited in DDBJ/EMBL/GenBank under the accession no. JTJB00000000. The version described in this paper is version JTJB01000000.
